# The crystal structures of the tri-functional *Chloroflexus aurantiacus* and bi-functional *Rhodobacter sphaeroides* malyl-CoA lyases and comparison with CitE-like superfamily enzymes and malate synthases

**DOI:** 10.1186/1472-6807-13-28

**Published:** 2013-11-09

**Authors:** Jan Zarzycki, Cheryl A Kerfeld

**Affiliations:** 1 Department of Biochemistry and Molecular Biology, Plant Research Laboratories, Michigan State University, Plant Biology Building, 612 Wilson Road, East Lansing, MI 48824, USA; 2 Department of Plant and Microbial Biology, University of California, Berkeley, CA 94720, USA; 3 Synthetic Biology Institute, University of California, Berkeley, CA 94720, USA; 4 Lawrence Berkeley National Laboratory, Berkeley, CA 94720, USA

**Keywords:** Malyl-CoA lyase, Malate synthase, Citrate lyase, CitE

## Abstract

**Background:**

Malyl-CoA lyase (MCL) is a promiscuous carbon-carbon bond lyase that catalyzes the reversible cleavage of structurally related Coenzyme A (CoA) thioesters. This enzyme plays a crucial, multifunctional role in the 3-hydroxypropionate bi-cycle for autotrophic CO_2_ fixation in *Chloroflexus aurantiacus*. A second, phylogenetically distinct MCL from *Rhodobacter sphaeroides* is involved in the ethylmalonyl-CoA pathway for acetate assimilation. Both MCLs belong to the large superfamily of CitE-like enzymes, which includes the name-giving β-subunit of citrate lyase (CitE), malyl-CoA thioesterases and other enzymes of unknown physiological function. The CitE-like enzyme superfamily also bears sequence and structural resemblance to the malate synthases. All of these different enzymes share highly conserved catalytic residues, although they catalyze distinctly different reactions: C-C bond formation and cleavage, thioester hydrolysis, or both (the malate synthases).

**Results:**

Here we report the first crystal structures of MCLs from two different phylogenetic subgroups in apo- and substrate-bound forms. Both the *C. aurantiacus* and the *R. sphaeroides* MCL contain elaborations on the canonical β_8_/α_8_ TIM barrel fold and form hexameric assemblies. Upon ligand binding, changes in the C-terminal domains of the MCLs result in closing of the active site, with the C-terminal domain of one monomer forming a lid over and contributing side chains to the active site of the adjacent monomer. The distinctive features of the two MCL subgroups were compared to known structures of other CitE-like superfamily enzymes and to malate synthases, providing insight into the structural subtleties that underlie the functional versatility of these enzymes.

**Conclusions:**

Although the *C. aurantiacus* and the *R. sphaeroides* MCLs have divergent primary structures (~37% identical), their tertiary and quaternary structures are very similar. It can be assumed that the C-C bond formation catalyzed by the MCLs occurs as proposed for malate synthases. However, a comparison of the two MCL structures with known malate synthases raised the question why the MCLs are not also able to hydrolyze CoA thioester bonds. Our results suggest the previously proposed reaction mechanism for malate synthases may be incomplete or not entirely correct. Further studies involving site-directed mutagenesis based on these structures may be required to solve this puzzling question.

## Background

**Figure 1:**
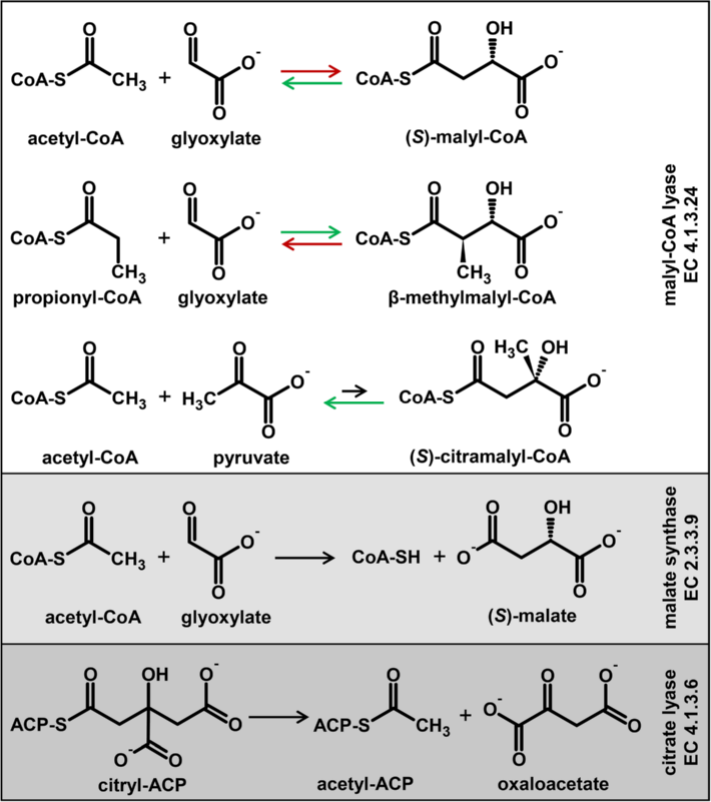
**Overview of reactions catalyzed by malyl-CoA lyases, the β-subunit of citrate lyase, and canonical malate synthases.** Green arrows represent reactions catalyzed by the malyl-CoA lyase in the 3-hydroxypropionate bi-cycle of *C. aurantiacus*. Red arrows represent reactions catalyzed by the malyl-CoA lyase in the ethylmalonyl-CoA pathway of *R. sphaeroides*. Note that all reactions catalyzed by malyl-CoA lyases are reversible. EC numbers for the different enzymes are provided.

Enzymes of the CitE-like superfamily are widely distributed among Bacteria, but can also be found in Archaea and Eukaryota. However, only very few of these enzymes have been biochemically characterized. The true CitE is the β-subunit of the ATP-independent citrate lyase, which consists of three different subunits [1,2] and the corresponding genes are part of the *citC*DEF(X)G operon [3,4]. The ATP-independent citrate lyase is only found in prokaryotes and is important for the anaerobic fermentation of citrate [1]. Its γ-subunit (CitD) functions as an acyl-carrier-protein (ACP) and contains a CoA derivative as prosthetic group [4-6]. The α-subunit (CitF) functions as an acyl-transferase and is responsible for the formation of a citryl-ACP intermediate [2]. CitE, the β-subunit, cleaves the citryl-moiety into oxaloacetate and acetyl-ACP (Figure 1) [2]. Other CitE-like enzymes are encoded by “stand alone” genes or genes that are part of clusters unrelated to the citrate lyase operon [7]. Examples of CitE-like enzymes that have been biochemically characterized use free acyl-CoA thioesters instead of ACPs as substrates, including the malyl-CoA lyases [8-12], a malyl-CoA thioesterase [11], and haloarchaeal forms of a malate synthases [13-15]. All of these enzymes require divalent metal ions, Mg^2+^ or Mn^2+^, for catalysis.

Malyl-CoA lyases are promiscuous enzymes that accept a variety of substrates and can catalyze the reversible aldol condensation of CoA thioesters like acetyl-CoA or propionyl-CoA with 2-oxoacids like glyoxylate or pyruvate [9-11]. MCLs are known to function in different pathways of central carbon metabolism in Bacteria and Haloarchaea. The very first MCLs were described in *Methylobacterium extorquens* AM1 (formerly *Pseudomonas* sp. AM1) [12,16] and *Aminobacter aminovorans* (formerly *Pseudomonas* sp. MA) [17]. In *Methylobacterium* an MCL takes part in the serine cycle, which allows the assimilation of C_1_ compounds such as methanol, formate, and formaldehyde into biomass [18]. In this case, MCL is responsible for the cleavage of (*S*)-malyl-CoA into glyoxylate and acetyl-CoA [16].

Subsequently it was discovered that in *C. aurantiacus* an MCL (MCLC) catalyzes three different steps in the 3-hydroxypropionate bi-cycle for autotrophic CO_2_ fixation [9,10,19]. This tri-functional enzyme cleaves (*S*)-malyl-CoA into acetyl-CoA and glyoxylate, combines glyoxylate with propionyl-CoA to β-methylmalyl-CoA, and finally also cleaves (*S*)-citramalyl-CoA into acetyl-CoA and pyruvate (Figure 1). This pathway is garnering considerable attention for biotechnological applications [20,21] because it is unique among the known CO_2_ fixing pathways in that the constituent enzymes are insensitive to oxygen [10]. Moreover, the whole bi-cyclic CO_2_ fixation strategy is metabolically streamlined; it comprises 19 chemical reaction steps but involves only 13 enzymes because several multifunctional enzymes are employed [10]. The tri-functionality of the MCLC underscores its key role for this pathway.

An MCL was also functionally characterized in *Rhodobacter capsulatus* and *R. sphaeroides*[8,11], which belong to a group of organisms that lack isocitrate lyase. Therefore, they are unable to use the glyoxylate bypass to assimilate acetyl-CoA or other substrates that enter central carbon metabolism at the level of acetyl-CoA. Instead, they use the ethylmalonyl-CoA pathway [22] for the assimilation of acetyl-CoA. In the ethylmalonyl-CoA pathway, characterized in the *R. sphaeroides,* the MCL (MCLR) is bifunctional; it catalyzes the cleavage of β-methylmalyl-CoA and the synthesis of malyl-CoA [11]. Interestingly, both MCLR and MCLC essentially catalyze the same reactions, but function in opposite directions in the ethylmalonyl-CoA pathway and the 3-hydroxypropionate bi-cycle, respectively (Figure 1).

Furthermore, MCL-like enzymes were found in Haloarchaea like *Haloarcula marismortui*, which lacks isocitrate lyase as well as enzymes that are required to establish the ethylmalonyl-CoA pathway. Nevertheless, it is still able to grow on acetate as the sole carbon source. It was recently demonstrated that these organisms employ yet another unique pathway for acetyl-CoA assimilation, the so called methylaspartate cycle [15]. This cyclic pathway makes use of two MCL-like enzymes, one of which seems to be optimized for the cleavage of β-methylmalyl-CoA into propionyl-CoA and glyoxylate (HaloMCL), whereas the other one acts like a malate synthase. The crystal structure of the homologous haloarchaeal malate synthase (HaloMS) from *Haloferax volcanii* was solved recently [23]. Although HaloMS shows only very low amino acid sequence identity (10 to 23%) to other malate synthases [23], all key catalytic residues in the active site are conserved.

**Figure 2:**
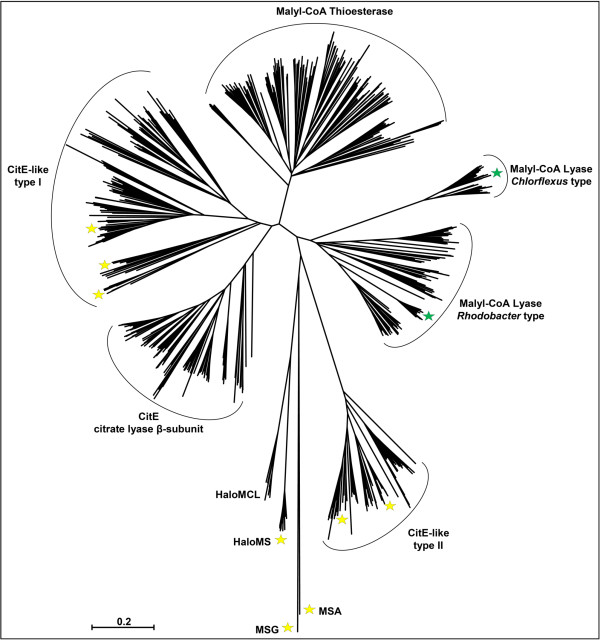
**Neighbor Joining Tree of the CitE-like superfamily of enzymes.** Canonical malate synthases of *E. coli* (MSG and MSA) serve as outgroup. Green stars represent crystal structures that were solved during this study. Yellow stars represent publically available structures of CitE-like enzymes and malate synthases (see Table 1). No structures are available for the true β-subunit of the ATP-independent citrate lyase (CitE). All types of known malyl-CoA lyases belong to phylogenitically distinct clusters, respectively. The malyl-CoA thioesterase of *R. sphaeroides* is also member of a separate sub-group of enzymes. Haloarchaeal malate synthase (HaloMS) is closely related to enzymes that constitute the haloarchaeal form of a malyl-CoA lyase (HaloMCL). The scale bar corresponds to the number of amino acid substitutions per site.

While several different members of the CitE-like superfamily of enzymes and the related malate synthases have been structurally characterized (Table 1, Figure 2), until now, no structures were available for enzymes of the phylogenetic subgroups that harbor malyl-CoA lyases. We determined the crystal structures of the phylogenetically distinct MCLs (Figure 2) of *C. aurantiacus* and *R. sphaeroides*, with and without bound substrates/ligands. The two MCLs share the common fold of a central TIM-barrel with small elaborations, as well as an additional C-terminal domain. In both cases the oligomeric state constitutes a dimer of trimers. The MCLC, however, is more compact than the MCLR, with a larger buried surface area between the two trimers. The trimeric assembly itself is a prerequisite for the catalytic activity of the MCLs as well as other CitE-like enzymes. This is due to a domain swap of the C-terminal domain that functions as a lid over the active site of the respective neighboring subunit. We observed different conformational states of this lid domain for both MCLs concomitant with substrate binding. Structural comparison of the MCLs with malate synthases provides hints as to what governs their substrate specificities and whether previously postulated reaction mechanisms for malate synthases also apply to MCLs.

**Table 1:** Enzymes used for phylogenetic and structural analyses

**Enzyme**	**Organism**	**GenBank Accession**	**PDB ID**
Malyl-CoA lyase	*Chloroflexus auranticus* OK-70-fl	AGR55786	4L7Z, 4L80
Malyl-CoA lyase	*Rhodobacter spaeroides* 2.4.1	ACJ71673	4L9Y, 4L9Z
Haloarchaeal malyl-CoA lyase	*Haloarcula marismortui*	YP_135395	-
CitE-like (RipC)	*Yersinia pestis* KIM10+	NP_669690	3QLL
CitE-like	*Pseudomonas aeruginosa* PAO1	NP_249574	-
CitE-like	*Mycobacterium tuberculosis* H37Rv	NP_217014	1U5V, 1U5H, 1Z6K
CitE-like	*Ralstonia eutropha* JMP134	YP_298346	3QQW, 3IUZ
CitE-like	*Deinococcus radiodurans* R1	NP_294964	1SGJ
CitE-like	*Burkholderia xenovorans* LB400	YP_552446	3R4I
CitE	*Klebsiella pneumonia* MGH 78578	YP_001333726	-
Haloarchaeal malate synthase	*Haloferax volcanii* DS2	YP_003536009	3OYZ, 3OYX, 3PUG
Malate synthase G	*Escherichia coli* K-12	NP_417450	1P7T
Malate synthase A	*Escherichia coli* K-12	NP_418438	3CUZ, 3CV1, 3CV2

## Results

### Structure determination of MCLC

The recombinant MCLC was purified from *E. coli* cell extracts. During gel filtration MCLC eluted at a molecular weight of 228 ± 15 kDa. This is consistent with a previously reported hexameric oligomerization state [9], because one monomer of the recombinant enzyme has a calculated molecular weight of 38.4 kDa. Activity of the purified enzyme was routinely confirmed by a spectrophotometric assay monitoring the formation of β-methylmalyl-CoA from propionyl-CoA and glyoxylate (see Methods).

**Figure 3:**
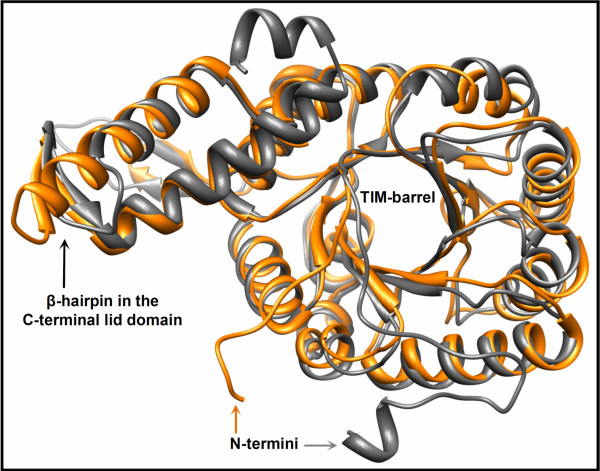
**Overlay of single subunits of both types of malyl-CoA lyases in the closed conformation.** MCLC (PDB 4L80) is colored grey. MCLR (PDB 4L9Y) is colored in orange. The rmsd between 267 Cα pairs is 0.96 Å. Secondary and tertiary structures are well conserved in both isoenzymes. The only major difference is in the orientation and lengths of the N-termini. Moreover, one of the helices that form the C-terminal lid domain is slightly shorter in MCLC compared to MCLR, whereas there is an additional small helix in the C-terminus of MCLC.

**Figure 4:**
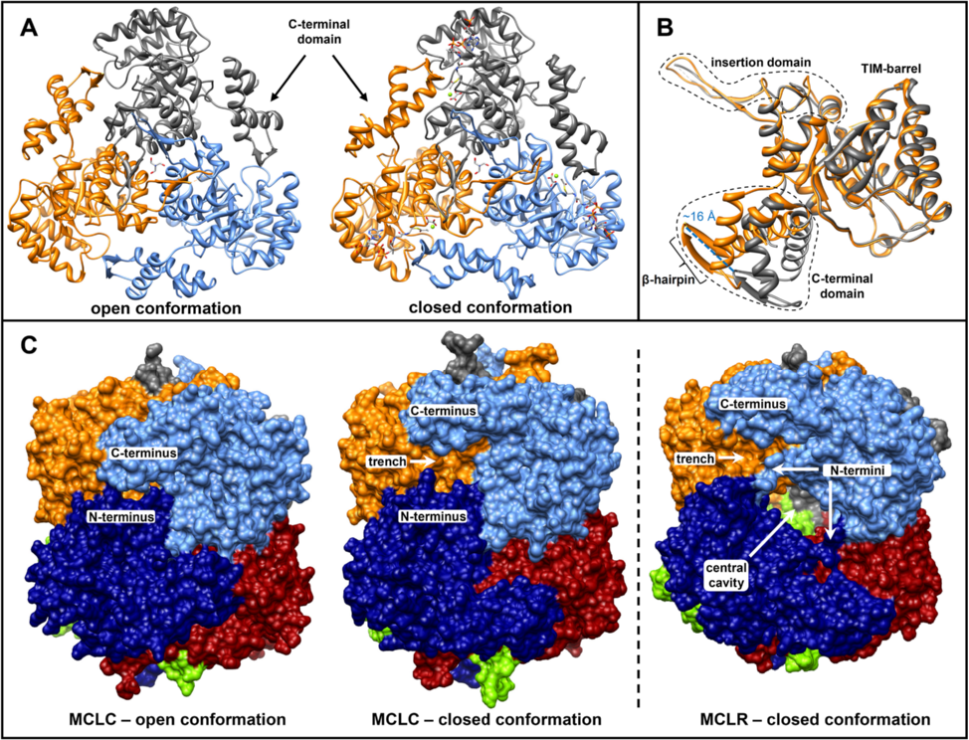
**Changes in MCL structures resulting from ligand binding. A)** Trimers of the MCLC structure in the open (PDB 4L7Z) and closed conformation (PDB 4L80) without and with bound substrates; respectively. The view is from the top along the 3-fold rotation axis. A Tris molecule is positioned at this axis buried in the protein. Ligands are depicted as stick models. **B)** An overlay of monomers of the MCLC structures in the closed form (orange) and open form (grey). The C-terminal lid domain is rotated about 30° resulting in a shift of approximately 16 Å at its extremity. **C)** Comparison of quaternary structures. Trenches at the surface that are present in the closed conformation between the N-termini and the C-terminal lid domains are completely covered by the lid domain in the open conformation. Therefore, the N-termini seem to limit the vertical movement of the lid domain. The different orientation and reduced size of the N-termini in MCLR are responsible for an opening in the hexameric assembly that allows access to a central cavity. A similar cavity is also present in MCLC, but the access is obstructed.

In the absence of substrates recombinant MCLC crystallized in the orthorhombic space group *P* 2 2_1_ 2_1_ with one hexamer per asymmetric unit (AU). The crystals diffracted to a resolution of 2.5 Å and the structure was solved using the molecular replacement method. The resulting model (PDB 4L7Z) comprised nearly the full length of all six polypeptide chains, starting at Arg2 and ending with Leu348 (native C-terminal). Only 4 to 6 residues were not built in a loop region (residues 210 – 215) of each chain due to the lack of sufficient electron density. As with other members of the CitE-like superfamily [7,24] and malate synthases [23,25-29], the core of the MCLC monomer constitutes a β_8_/α_8_ TIM-barrel (Figure 3). In addition to the central TIM-barrel there is a C-terminal domain (starting at Phe287) that comprises three α-helices of which two are connected by a β-hairpin (residues 310–317) (Figure 3). This C-terminal domain extends to the neighboring subunit (Figure 4).

Soaking attempts with the MCLC substrate propionyl-CoA resulted in rapid dissolution of the crystals. Therefore, attempts were made to crystallize MCLC in the presence of substrates. Using different crystallization buffers (see Methods) we were able to obtain crystals in the tetragonal space group *P*4_3_, when propionyl-CoA, oxalate and magnesium ions were present. Oxalate was chosen instead of glyoxylate because of the structural similarity between the two compounds, and because oxalate also acts as an inhibitor of malyl-CoA lyase. The crystals contained one hexamer per AU and diffracted to 2.0 Å. The resulting structure (PDB 4L80) comprised the complete peptide chains for all six subunits with the exception of residues 211–213 in two of the six chains, as well as the two C-terminal residues (Gly347 and Leu348) from a third chain. Interestingly, the C-terminal domain in this structure was shifted about 16 Å at its extremity (α-carbon of Gly314) resulting in the closure of the putative active site of the neighboring subunit (Figure 4). Therefore the C-terminal domain appears to serve as a flexible lid. Moreover, in all 6 of these putative active sites electron density was observed that allowed modeling of propionyl-CoA, oxalate and Mg^2+^. Notably, the conformational change of the C-terminal lid domain may explain why the native crystals dissolved upon soaking with substrate. Statistics for the two different crystal structures are given in Table 2.

**Table 2:** **Data collection and refinement statistics of malyl-CoA lyase of *C. aurantiacus***

**PDB ID**			**4L7Z**			**4L80**		
**Ligands**			**Tris**			**propionyl-CoA, oxalate, Mg**^**2+**^**, Tris**		
**Space group (No.)**			***P*** **2 2**_**1**_**2**_**1**_**(18)**			***P*** **4**_**3**_**(78)**		
**Conformation**			**all subunits open**			**all subunits closed**		
Unit cell dimensions								
a	b	c [Å]	96.6	157.8	168.11	102.2	102.2	204.2
α	β	γ [°]	90.0	90.0	90.0	90.0	90.0	90.0
Resolution [Å]			38.5 - 2.5			37.9 - 2.0		
Number of observations								
total			578,015			704,447		
unique			88,943			138,647		
redundancy			6.5			5.1		
Complete (last shell) [%]			99.6 (97.3)			99.8 (99.2)		
*I*/*σ*(*I*) (last shell)			10.9 (2.4)			15.1 (2.8)		
*R*_merge_ (last shell)			0.155 (0.685)			0.106 (0.637)		
Refinement								
*R*_work_			0.190			0.172		
*R*_free_			0.236			0.201		
RMSD bond lengths [Å]			0.002			0.006		
RMSD bond angles [°]			0.597			0.910		
mean B-factor (ligands) [Å^2^]			21.7 (13.6)			28.3 (35.8)		
Ramachandran								
favored [%]			97.44			96.51		
allowed [%]			2.56			3.39		
outliers [%]			0.00			0.10		

### Structure determination of MCLR

The recombinant MCLR was also initially crystallized in the absence of substrates. The crystals grew in space group *P* 1 2_1_ 1 with one hexamer per AU. During gel filtration the His_10_-tagged MCLR eluted with a molecular weight of 220 ± 15 kDa, consistent with a hexameric assembly (36.8 kDa per monomer), as was previously reported [11]. Activity of the purified MCLR was also routinely confirmed using the spectrophotometric assay. We also verified MCLR’s ability to catalyze the reversible formation of citramalyl-CoA from acetyl-CoA and pyruvate, because that has not been tested before [11]. MCLR was incubated with acetyl-CoA in the presence of (200-fold excess) pyruvate. The formation of citramalyl-CoA was confirmed by reversed phase HPLC analysis of the reaction mixture;the reaction reached an equilibrium of about 1 : 2.5 (citramalyl-CoA: acetyl-CoA). For comparison, it was reported for MCLC that this reaction reaches an equilibrium of about 150 when pyruvate was used in only 10-fold excess over acetyl-CoA [10].

Diffraction of the crystals reached a resolution of 2.1 Å and the structure was solved by molecular replacement. In the final model (4L9Y) each chain begins at Ser2, whereas varying numbers of residues had to be excluded from the C-termini. Three of the six chains lacked electron density for the entire C-terminal lid domains and were modeled only to Pro265 or Ser266. For the other 3 chains it was possible to model the lid domains with the exception of only the terminal 1, 3, or 8 residues.

Despite repeated attempts, we were not able to obtain crystals of an apo-enzyme with electron density simultaneously present for all 6 of the C-terminal lid domains. Notably, of the three lid domains that could be modeled only two were in the closed conformation. In the other one the β-hairpin structure that is depicted in Figure 3 was not developed, and the domain was shifted about 8 Å at its extremity (α-carbon of Gly295). The shift of the C-terminal lid domain is similar to what was observed for the MCLC structures with and without bound substrates. Interestingly, in the two subunits where the lid domains were in the closed conformation, the active sites contained electron density that allowed modeling of glyoxylate molecules together with magnesium ions. Nevertheless, Mg^2+^ could also be fitted into the 4 open active sites, whereas glyoxylate was replaced by water molecules in these subunits. Although glyoxylate was not intentionally present, it was likely carried over from *E. coli* cell extracts during enzyme purification.

Soaking of these crystals with propionyl-CoA resulted in the additional occupancy of one of the two closed active sites with the CoA thioester. Additional soaking attempts resulted either in dissolving crystals or only very weak electron density for the ligands. Hence, crystals were grown in the presence of propionyl-CoA, oxalate and magnesium ions. The new buffer conditions typically resulted in crystals of the rhombohedral space group *R* 3 2 with one monomer per AU. These crystals diffracted to a resolution of 2.2 Å. Although the C-terminal lid domain could be completely resolved and was in the closed conformation, the electron density for bound substrates was scant. However, the same crystallization conditions occasionally yielded a second type of crystals in the hexagonal space group *P* 6_1_, with one hexamer per AU. These crystals diffracted to a resolution of 2.0 Å. All six C-terminal lid domains were resolved and all of the active sites were in the closed conformation. Each active site was occupied by Mg^2+^, oxalate, and free CoA instead of propionyl-CoA. The polypeptide chains in the resulting model (PDB 4L9Z) comprised all residues from Ser2 or Phe3 through Met315 with only the C-terminal three residues missing. Electron density for the N-terminal His_10_-tags was also missing for each chain. The statistics for the two different MCLR crystal structures are given in Table 3.

**Table 3:** **Data collection and refinement statistics of malyl-CoA lyase of *R. sphaeroides***

**PDB ID**			**4L9Y**			**4L9Z**		
**Ligands**			**propionyl-CoA, glyoxylate, Mg**^**2+**^**, Cl**^**-**^			**CoA, oxalate, Mg**^**2+**^		
**Space group (No.)**			***P*** **1 2**_**1**_**1 (4)**			***P*** **6**_**1**_**(169)**		
**Conformations**			**open and closed subunits**			**all subunits closed**		
Unit cell dimensions								
a	b	c [Å]	80.2	144.0	94.2	221.5	221.5	96.3
α	β	γ [°]	90.0	112.8	90.0	90.0	90.0	120.0
Resolution [Å]			38.8 - 2.1			38.5 - 2.0		
Number of observations								
total			428,450			1,116,278		
unique			113,853			175,339		
redundancy			3.8			6.4		
Complete (last shell) [%]			99.6 (98.2)			98.3 (99.0)		
*I*/*σ*(*I*) (last shell)			15.8 (3.2)			11.7 (2.6)		
*R*_merge_ (last shell)			0.066 (0.448)			0.158 (0.744)		
Refinement								
*R*_work_			0.177			0.170		
*R*_free_			0.207			0.194		
RMSD bond lengths [Å]			0.003			0.006		
RMSD bond angles [°]			0.746			0.881		
mean B-factor (ligands) [Å^2^]			23.6 (32.2)			16.1 (25.7)		
Ramachandran								
favored [%]			97.61			97.97		
allowed [%]			2.27			2.03		
outliers [%]			0.12			0.00		

### Primary and tertiary structure comparison between the monomers of MCLR and MCLC

Although the amino acid sequence identity between MCLC and MCLR is relatively low (~ 37%), their tertiary structures are strongly conserved (Figure 3). The subunits of both enzymes comprise a central β_8_/α_8_ TIM-barrel with some insertions of small secondary structure elements. Both MCLs have mostly unordered N-termini (residues 2–29 in MCLC and 2–15 in MCLR), leading to the first β-strand of the TIM-barrel (Figure 3). However, the orientation and length of the N-termini differs between MCLC and MCLR. MCLR has N-terminal His_10_-tag, which could not be modeled due to the lack of electron density. We cannot discount the possibility that the His-tags may have had an influence on the orientation of the N-termini in MCLR.

Another elaboration of the TIM-barrel core common to both MCL structures is found after the sixth β-strand (Figure 4B). This insertion (residues 182–220 in MCLC and 167–197 in MCLR) comprises an additional α-helix leading into an unordered loop that connects to a β-hairpin loop structure. The corresponding β-strands in MCLC are connected by a larger hairpin loop that consists of 11 residues (Ala207-Pro217), whereas the corresponding turn in MCLR only comprises Asp192 and Gly193. These insertion domains appear to be present in all of the enzymes that cluster together with MCLC or MCLR in the phylogenetic tree (Figure 2), respectively (compare HMM-logos in Additional file 1: Figure S1 and Additional file 2: Figure S2).

In addition to the core TIM-barrel structure there is a C-terminal lid domain in both MCLs. These lid domains differ slightly in MCLC (residues 287–348) and MCLR (residues 264–318), but both comprise two α-helices that are connected by a β-hairpin (Figures 3 and 4). In MCLR the first of the two helices is slightly longer in comparison to MCLC, whereas MCLC possesses one additional short helix at the very end of the lid domain.

### MCLR and MCLC oligomeric state

Both types of MCLs are hexameric, composed of dimers of trimers (Figure 4). The average interface areas between adjacent subunits in the trimers of MCLC and MCLR (closed conformations) are similar, 2037 Å^2^ and 2154 Å^2^, respectively. The MCLC hexamer appears to be more compact than the MCLR (Figure 4C) with a calculated buried surface area at the dimer interface between two trimers of 4594 Å^2^ in MCLC but only 3173 Å^2^ in MCLR. The dimer interface between the trimers is expanded in MCLC mostly due its longer N-termini, which are in a different orientation than in the MCLR structures. The shorter N-termini in the MCLR hexamer also allow access to a central cavity between the trimers. A similar cavity is present in MCLC as well, but it is only accessible through very narrow pores that seem just wide enough to allow diffusion of water molecules. It is not clear if the central cavities in either enzyme serve a particular function.

The N-terminal amino acid sequence is highly conserved among the enzymes that cluster together with MCLC in the phylogenetic tree (Figure 2). In contrast, the N-terminus is not well conserved among enzymes of the MCLR cluster. However, the N-termini of both enzymes seem to limit the movement of the C-terminal lid domains in the hexameric assemblies (Figure 4C).

A second distinctive structural feature of the closed form of both MCLs is that the β-hairpin (Figures 3 and 4B) of the C-terminal lid domain provides a number of residues to close the active site. One of these residues (Asp318 in MCLC and Asp299 in MCLR) is presumed to take part in the first step of catalysis as proposed for the reaction mechanism of malate synthases [27,30], discussed below.

### Ligand binding

**Figure 5:**
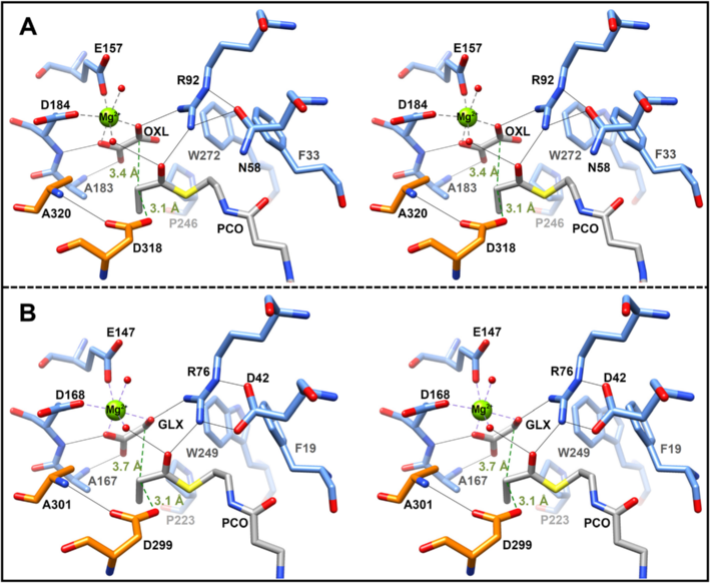
**Comparison of the active sites of both malyl-CoA lyases.** Ligands are colored in grey. Residues of the TIM-barrel are colored in blue, whereas residues of the C-terminal lid domains of the neighboring subunits are colored in orange. Important hydrogen bonds are depicted as thin black lines. Coordination of the Mg^2+^ ion is shown by thick grey broken lines. Distances between the reacting α-carbon of propionyl-CoA (PCO) to the proposed active aspartate residue and oxalate (OXL) or glyoxylate (GLX) are illustrated in green. **A)** Stereo view of the active site of MCLC (PDB 4L80). **B)** Stereo view of the active site of MCLR (PDB 4L9Y).

**Figure 6:**
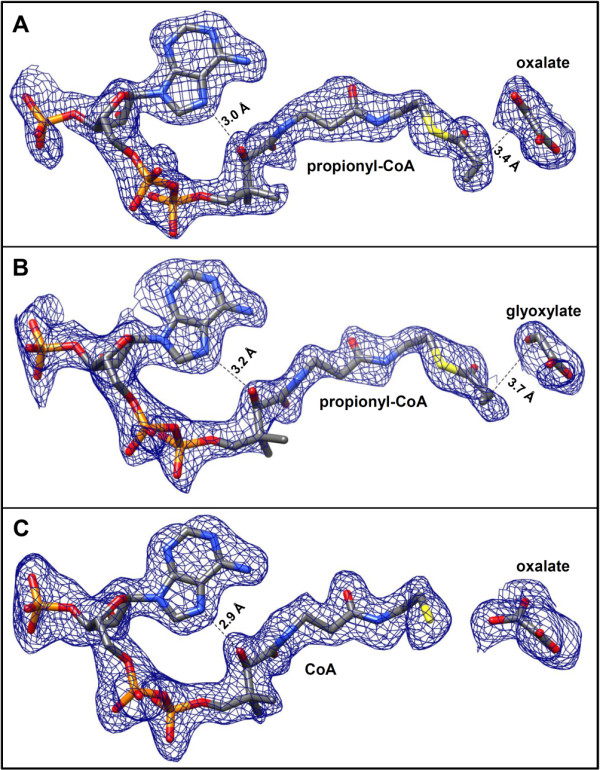
**Superpositions of *F***_**o**_**-*F***_**c **_**electron density simulated annealing omit maps on refined ligands.** The CoA moieties assume bent J-like conformations in the structures of MCLC and MCLR. Intramolecular hydrogen bonds between the adenosine rings and the pantetheine tails are indicated. **A)** Omit map at 2.5 σ for propionyl-CoA and oxalate bound in the active sites of the MCLC structure (PDB 4L80). The α-carbon of the propionyl moiety is in close proximity to oxalate (3.4 Å). **B)** Omit map at 2 σ for propionyl-CoA and glyoxylate modeled into one of the active sites of the MCLR structure (PDB 4L9Y). The α-carbon of the propionyl moiety is 3.7 Å from the carbonyl carbon of glyoxylate. **C)** Omit map at 2.0 σ for CoA and oxalate bound in six of the active sites in MCLR structure (PDB 4L9Z).

**Figure 7:**
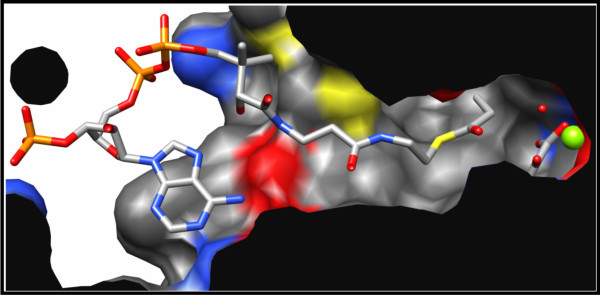
**Slabbed side view of the MCLC active site surface model in the closed conformation.** A pocket at the protein surface accommodates the adenosine moiety of propionyl-CoA, whereas its pantetheine moiety is threaded into the tunnel-like active site cavity. The Mg^2+^ ion (green) is located at the very end of the tunnel and is coordinated by oxalate. Surface colored by atom type: red (oxygen), blue (nitrogen), yellow (sulfur).

By crystallizing both types of MCLs in the presence of propionyl-CoA, oxalate, and Mg^2+^, we obtained structures in which all six active sites were closed off by the C-terminal lid domains of the respective neighboring subunits. Moreover, the electron density was sufficient to fit ligands into each active site. The Mg^2+^ ion was coordinated by glutamate and aspartate residues (MCLC: Glu157 & Asp184, MCLR: Glu141 & Asp168), as well as two oxygen atoms from oxalate and the oxygen atoms of two water molecules (Figure 5). The resulting octahedral shell around the bound magnesium ion resembles the ion binding observed in other CitE-like enzymes and different malate synthases [7,23,25,27,28]. The binding of the CoA-moiety is also very similar for MCLC, MCLR and the malate synthases. The adenosine moiety of CoA is fixed in a largely hydrophobic pocket on the surface of the TIM-barrel with only one or two hydrogen bonds formed between the adenine ring and carbonyl oxygen atoms of the protein backbone. In MCLC there is an additional hydrogen bond formed with the side chain of His32, a residue conserved only among MCLC related enzymes. Due to the bent J-like conformation of the CoA-moiety (Figure 6) an intramolecular hydrogen bond between the adenosine ring and the hydroxyl-group of the pantheteine is formed in both MCLs that is also reported for malate synthases [23,25,26]. The phosphate groups are coordinated by arginine, lysine, or histidine residues and the pantheteine tail is inserted (threaded) into the quite narrow and deep active site cavity (Figure 7) formed in the closed conformation.

**Figure 8:**
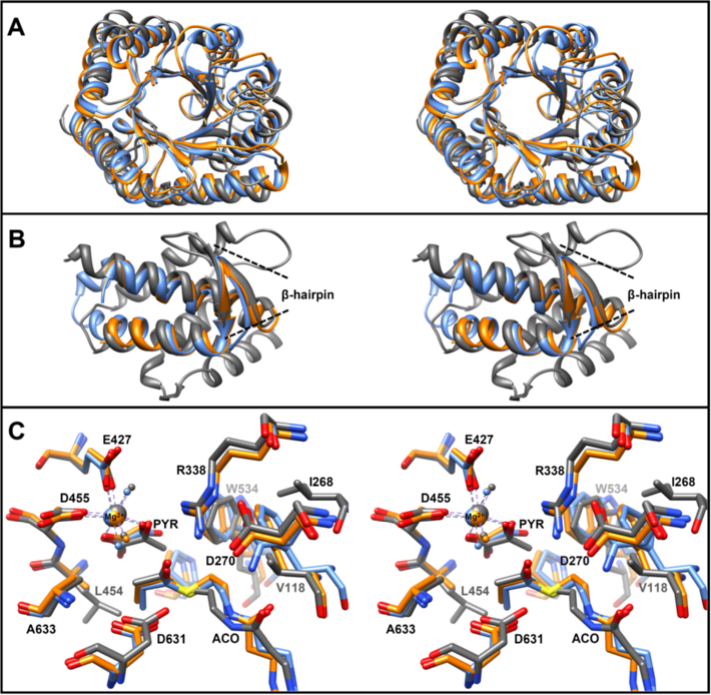
**Overlays of MCLC, MCLR, and MSGE. A)** Stereo view of a superposition of only the central TIM-barrel secondary structure elements of MCLC (PDB 4L80), MCLR (PDB 4L9Y), and malate synthase G of the *E. coli* (PDB 1P7T). MCLC (blue), MCLR (orange), MSGE (gray). The overall rmsd between 212 Cα pairs is 2.0 Å. **B)** Stereo view of a superposition of the C-terminal lid domains. The rmsd between 42 Cα pairs is 1.4 Å. Only two of the α-helices as well as their connecting β-hairpins are structurally conserved between the MCLs and malate synthases. **C)** Stereo view overlay of active site residues and bound ligands (rmsd is the same as in A). Propionyl-CoA and oxalate are bound in MCLC, propionyl-CoA and glyoxylate are bound in MCLR, whereas acetyl-CoA (ACO) and pyruvate (PYR) are bound in MSGE. The numbering of residues corresponds to MSGE. A positionally conserved alanine residue in all malyl-CoA lyases is substituted by Leu454 in MSGE; it may sterically hinder propionyl-CoA and β-methylmalyl-CoA binding.

In the MCLR crystal structure with all six C-terminal lid domains modeled, only free CoA (Figure 6C) was found instead of propionyl-CoA. It is known that CoA thioesters are more stable under acidic conditions and become hydrolyzed over time under alkaline conditions. The pH of the crystallization condition was 7.5, in contrast to pH 5.5 used to grow the MCLC crystals. Although we have also grown crystals in the presence of propionyl-CoA in different, slightly acidic conditions, we were not able to obtain other structures with bound propionyl-CoA. However, propionyl-CoA soaking attempts with the first type of MCLR crystals, which only allowed the modeling of three of the six C-terminal domains, were partly successful. These crystals already had magnesium and glyoxylate present in the two active sites that were in the closed conformation. After the additional soaking step, one of these closed active sites was also occupied by propionyl-CoA (Figures 5B & 8B).

In both MCLC structures (with and without bound substrates) additional electron density was observed at the 3-fold rotation axis in each trimer and was modeled as Tris molecules. These form hydrogen bonds with the side chain amide oxygens of Gln221 in each subunit of the trimer, as well as with backbone oxygen atoms of Asp222. The Tris molecules are buried within the protein and do not appear to be solvent accessible, which may indicate that the additional insertion domain (residues 182–220) found after the sixth β-strand of the TIM-barrel is able to undergo a conformational change.

## Discussion

### The structures of MCLC and MCLR and comparison to structures of malate synthases and CitE-like family enzymes

The malyl-CoA lyase structures reported here provide the first structures of two subgroups of the CitE-like superfamily of enzymes (Figure 2). Both MCLC and MCLR constitute dimers of trimers as their oligomeric state. The structures suggest that monomers of MCLC and MCLR cannot be catalytically active, because the C-terminal lid domain of each subunit in the trimer stretches out to the neighboring one and contributes active site residues. This is in contrast to the malate synthases of type A and G, which are structurally and functionally monomeric. Interestingly, the HaloMS was reported to have a trimeric or hexameric (dimer of trimers) assembly [23] as well. Therefore the HaloMS may be evolutionarily more closely related to the malyl-CoA lyases and other enzymes of the CitE-like superfamily, which have also been reported to be trimers [7,24], including the malyl-CoA thioesterase of *R. sphaeroides*[11].

Although the TIM-barrel is conserved in malate synthases, MCLs (Figure 8A), and other CitE-like enzymes, both malate synthase A (MSA) and malate synthase G (MSG) also possess an additional N-terminal domain [23,28] of about 90 and 115 residues, respectively, that folds around the TIM-barrel. Furthermore MSG has a rather large (~ 200 residues) domain insertion between TIM-barrel secondary structure elements. In contrast, MCLC and MCLR both have a smaller insertion of ~40 and ~30 residues after the sixth β-strand of the TIM-barrel, which appears to be specific to the MCLs, judging from structure and sequence comparisons with malate synthases and CitE-like enzymes. These additional domains are arranged around the three-fold axes on top of the trimers and are in contact with one another. However, there are also differences in the C-terminal domains of the MCLs in comparison to MSA, MSG, and HaloMS [23]. In MSA and MSG the domain consists of 5 α-helices and the β-hairpin. The β-hairpin is structurally conserved in all of the aforementioned enzymes, whereas the number of α-helices differs (Figure 8B). In contrast to the canonical malate synthases, this lid domain in the MCLs closes off the active site of the neighboring subunit in the trimers and not of its own TIM-barrel. This is probably also the case in the HaloMS, because a region of more than 40 residues is missing from its crystal structures (PDB 3PUG, 3OYX, 3OYZ) that would connect the TIM-barrel with the C-terminal domain. Although this domain was modeled as if it were covering the active site of the same subunit, it is possible that it actually extends to the neighboring subunit as explained by Bracken *et al.*[23].

### Movement of the C-terminal lid domain

Rotations of the C-terminal lid domains of ~30° in MCLC (Figure 4B) and ~18° in MCLR relative to their TIM-barrel cores were observed. In both structures the bending region is located in a short linker that connects the last helix of the TIM-barrel with the first helix of the lid domain. In both cases the linkers start with a phenylalanine residue (Phe286 and Phe263, respectively). The bending of the chains progresses through residues Ser287 and Pro288 in MCLC and through Thr264 and Pro265 in MCLR (in Additional file 1: Figure S1 and Additional file 2: Figure S2). The phenylalanine and proline in these linkers appear to be well conserved within the CitE-like superfamily of enzymes. It is therefore likely that a similar conformational change can occur in these enzymes as well.

Interestingly, there is much less interaction of each TIM-barrel with the neighboring C-terminal domains in the open conformation assembly; the reduction in surface area is ~500 Å^2^ in both MCLC and MCLR. The reduced interaction of the lid domain with the TIM-barrel in the open conformation probably leads to increased flexibility and multiple conformations. This may be the reason why the electron density for the lid domain beyond the bending region was weak or non-existent for three of the subunits in the MCLR structure that was soaked with propionyl-CoA. Furthermore, the average temperature factors of residues in the lid domains are about two times higher than those of the TIM-barrel residues in both MCLs, underscoring their flexibility. This is also consistent with the previously reported structures of RipC of *Y. pestis* (PDB 3QLL) and another CitE-like enzyme of *M. tuberculosis* (PDB 1U5H). Both of these enzymes belong to the type 1 subgroup in the phylogentic tree of the CitE-like superfamily (Figure 2). In both these cases the lid domains could not be modeled due to the lack of electron density [7,24].

The mobility of the C-terminal domain may have an effect on substrate binding. As mentioned above, for MCLR crystals grown in the absence of added substrates, electron density (modeled as glyoxylate) was only observed in the two subunits with completely closed active sites. After an additional soaking step with propionyl-CoA, electron density for the CoA thioester was found in one of these two sites.

The only other observed conformational changes in the MCLC and MCLR structures upon substrate binding are associated with the movement of the C-terminal domains. The changes occur in a loop consisting of residues 192–203 in MCLC and residues 174–187 in MCLR. This loop belongs to the additional MCL-specific small insertions mentioned earlier. The loop interacts, predominantly through hydrogen bonds and bridging waters, with the C-terminal domains of the neighboring subunits in the closed conformation. In enzymes that cluster together with MCLC in the phylogenetic tree (Figure 2) the corresponding loop region is extremely well conserved. However, the same loop region in the MCLR is only conserved in more closely related enzymes and not throughout the whole cluster of MCLR-like lyases. However, this cluster comprises enzymes that share less than 40% amino acid sequence identity with MCLR. In contrast, enzymes that cluster together with the MCL of *C. aurantiacus* are much more closely related to one another, with amino acid identities of at least 57% in pairwise alignments.

Mobility of the C-terminal lid domain was also proposed for malate synthases (MSA and MSG) [27], but not observed in crystal structures. The C-terminal domain is much larger in MSA and MSG than in CitE-like enzymes, which include the haloarchaeal malate synthase (HaloMS). However, small angle X-ray scattering and circular dichroism experiments with malate synthases from baker’s yeast and maize [31-33] suggested a conformational change within the enzymes upon substrate binding.

### Active sites and substrate binding

The C-terminal lid domains seem to play crucial roles in the interaction with the substrates. Most of the active site residues contributed by the lid domains appear to be involved in the binding of the pantetheine moiety of the CoA thioester substrate. Moreover, an aspartate residue (Asp318 in MCLC and Asp299 in MCLR) located in the C-terminal domain structurally aligns with a putative catalytic aspartate in the structures of HaloMS, MSA and MSG (Figure 8C). Despite the low amino acid sequence identity between both MCLC and MCLR, most of the residues involved in the formation of the active site and substrate binding are conserved, including all of the putative catalytic residues (Figures 5 and 8C). The residues Arg92 and Asp318 in MCLC correspond to Arg76 and Asp299 in MCLR; they are conserved in the entire CitE-like superfamily as well as in the malate synthases. The only exception was found in the enzymes that cluster together with the malyl-CoA thioesterase of *R. sphaeroides* in the phylogenetic tree (Figure 2). A conserved glutamate residue is present instead of aspartate in these enzymes. The glutamate and aspartate residues that coordinate the Mg^2+^ ion (Glu157 & Asp184 in MCLC, Glu141 & Asp168 in MCLR) are also absolutely conserved among CitE-like superfamily enzymes and the malate synthases. The same is true for the residues Glu60 and Asp61 in MCLC (corresponding to Glu44 and Asp45 in MCLR), which form hydrogen bonds with the two water molecules that also coordinate the Mg^2+^ ion. The remaining two sites of the octahedral Mg^2+^ coordination sphere are occupied by oxygen atoms of the respective bound carbonic acid, which is oxalate or glyoxylate in the structures of the MCLs. This Mg^2+^ coordination is consistent with available structures of malate synthases (compare Figure 8C).

Only a minor difference can be observed between the active sites of MCLC and MCLR. The conserved arginine residue in MCLC (Arg92) forms a hydrogen bond with a neighboring asparagine (Asn58). This asparagine residue is 100% conserved among the lyases that cluster together with MCLC in the phylogenetic tree (Figure 2). In MCLR this residue is replaced by an aspartate (Asp42), which also forms hydrogen bonds with the corresponding Arg76 (Figure 5). The aspartate residue, however, is absolutely conserved for the CitE-like superfamily enzymes, as well as among malate synthases. The positioning of the arginine residue, however, does not seem to be affected by the Asp/Asn substitution in the MCLC. However, it appears that the hydrogen bonding is generally important for the correct orientation of the arginine and therefore the binding of the substrates. This is also evident from mutational studies on the MSG from *E. coil*, where the positionally conserved arginine residue was replaced by lysine; this resulted in a substantially reduced catalytic activity (6.6% of wild type level) as well as in a 10-fold increase of the *K*_m_ value for acetyl-CoA [25].

### Comparison of reaction mechanisms and substrate specificities of MCLR, MCLC and malate synthases

**Figure 9:**
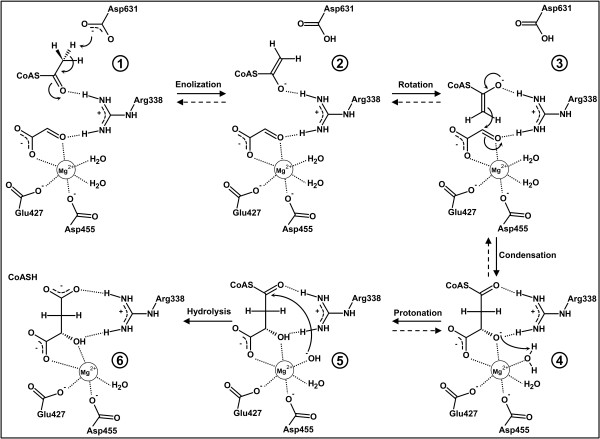
**Previously proposed reaction mechanism for malate synthase (adapted from Howard *et al*., 2000).** Residue numbers correspond to malate synthase G of *E. coli*. Asp631 abstracts a proton from the α-carbon of acetyl-CoA **(1)**. The enolate intermediate **(2)** performs a nucleophilic attack on the carbonyl carbon of glyoxylate **(3)**. The oxyanion in the newly formed malyl-CoA intermediate **(4)** is stabilized by the positive charges of Mg^2+^ and Arg338 [27]. The exact course of the following CoA thioester hydrolysis is not known. One of the water molecules coordinating the magnesium ion may be responsible for the protonation of the oxyanion resulting in the formation of a hydroxyl-anion **(5)**, which subsequently attacks the CoA thioester bond [30] and facilitates the formation of malate and free CoA **(6)**. The dashed arrows represent reversed reactions that have to be catalyzed by malyl-CoA lyases if this proposed reaction mechanism is correct.

A reaction mechanism for malate synthase (Figure 9) was proposed by Howard et al. [27] for the *E. coli* MSG (MSGE). Asp631 acts as a base and abstracts a proton from the α-carbon of acetyl-CoA [34,35]. The importance of this aspartate residue was demonstrated by mutation to asparagine, which led to the complete loss of enzymatic activity [25]. The negative charge of the enolate that is created upon proton abstraction from acetyl-CoA is stabilized by Arg338, which also interacts with the carbonyl oxygen of glyoxylate. After rotation of the enolate intermediate, a nucleophilic attack on the carbonyl carbon of glyoxylate leads to the formation of a new carbon-carbon bond and an oxyanion (Figure 9). The oxyanion is stabilized by the positive charges of Mg^2+^ and Arg338. Unfortunately, it is not quite clear how the CoA thioester hydrolysis proceeds. It was proposed that one of the two water molecules coordinating the Mg^2+^ ion may be responsible [30]. However, the removal of this water from the octahedral coordination sphere of the magnesium ion is energetically unfavorable [36]. Furthermore, these two water molecules are also present in the structures of the malyl-CoA lyases, which do not exhibit any detectable thioester hydrolysis activity. Therefore, another water molecule may be responsible for the CoA thioester hydrolysis step. Although the active sites of malate synthases and MCLs appear to be highly conserved, their respective reaction and substrate specificities differ (see Figure 1 for comparison). Both types of enzymes catalyze aldol reactions, which is completely reversible in the MCLs, but not in the malate synthases due to the additional CoA thioester hydrolysis step. However, the aldol reaction and the thioester hydrolysis have to occur independently of one another if a product like malyl-CoA is to be released from the active site. It is safe to assume that the aldol reaction in the MCLs also proceeds via the formation of an enolate intermediate (Figure 9), as was proposed for malate synthases. However, in order for this reaction to be reversible, as it is in the MCLs (Figure 1), a proton has to be abstracted from the hydroxyl group of the respective CoA thioester substrates like malyl-CoA, β-methylmalyl-CoA, or (*3S*)-citramalyl-CoA. Because this hydroxyl group is coordinating the Mg^2+^ ion its p*K*_a_ value would be considerably lowered. The hydrogen of the hydroxyl group would point away from the magnesium ion towards the conserved arginine residue (Arg92 in MCLC, Arg76 in MCLR), which seems to form a hydrogen bond with this hydroxyl group. This makes the arginine residue a likely candidate for the proton abstraction from the hydroxyl group of malyl-CoA and similar substrates, despite its usually high p*K*_a_ value. Although it is fairly rare that an arginine residue acts as a base in proton abstraction reactions, examples are known [37]. After the carbon-carbon bond cleavage, the enolate intermediate has to be neutralized by proton donation from the conserved aspartate residue (Asp318 in MCLC, Asp299 in MCLR). Therefore, both the Arg and Asp residues must be available in deprotonated and protonated forms to allow the aldol reaction to proceed in either direction. Unfortunately, it is not clear from the MCL crystal structures and comparisons to malate synthases (including HaloMS) why MCLs do not also act as thioesterases. As mentioned above, the CoA thioester hydrolysis may depend on another solvent water molecule, which could be present at the malyl-CoA intermediate step (see Figure 9, intermediate 4) during catalysis in malate synthases, but not in malyl-CoA lyases. Unfortunately, there are no structures available that have captured the malyl-CoA intermediate or a bound analog; there are only structures containing either acetyl-CoA or free CoA.

Nevertheless, within the two MCL clusters in the phylogenetic tree (Figure 2) an alanine residue (Ala183 in MCLC and Ala167 in MCLR) is 100% conserved (in Additional file 1: Figure S1 and Additional file 2: Figure S2), whereas it is replaced by Leu454 in MSGE (Figure 8C) and Trp277 in MSA of *E. coli*. These larger side chains likely prevent the efficient binding of propionyl-CoA or β-methylmalyl-CoA by the malate synthases. In the overlay of the active sites (Figure 8C) the distances between Leu454 of MSGE and the terminal carbons of the propionyl-CoA molecules in MCLC and MCLR are 2.1 Å and 2.3 Å, respectively. Interestingly, these residues correspond to a Val191 in the HaloMS, which is conserved among haloarchaeal malate synthases and haloarchaeal MCLs. Valine is just small enough to allow propionyl-CoA binding, which may explain why HaloMS is still able to catalyze the formation of β-methylmalyl-CoA [15]. Why the CoA thioester bond in β-methylmalyl-CoA is not hydrolyzed by HaloMS cannot be explained at this point.

Furthermore, MCLC is known to efficiently catalyze the cleavage of (*S*)-citramalyl-CoA into acetyl-CoA and pyruvate, whereas the reverse reaction of citramalyl-CoA synthesis was only reported in high excess concentrations of pyruvate [10]. Malate synthases are obviously able to bind pyruvate together with acetyl-CoA, as observed in the crystal structures of MSGE (PDB 1P7T) and HaloMS (PDB 3OYZ). However, in the HaloMS structure the methyl group of pyruvate forms close contacts with Pro231 and Trp257 [23]. An expansion of the active site was observed in the HaloMS structure with bound pyruvate and acetyl-CoA [23], compared to the structure that only had glyoxylate bound (PDB 3OYX). This may be the reason why addition of pyruvate to acetyl-CoA is unfavorable in HaloMS as well as in the MCLs, where both the proline and the tryptophan residues are conserved (Figure 5 & 8C). On the other hand, the geometry at the carbonyl-carbon of pyruvate is planar, whereas the geometry at the corresponding carbon in citramalyl-CoA is tetrahedral. Therefore, the methyl group of citramalyl-CoA would assume a different position further away from the proline and tryptophan residues, which may favor binding of citramalyl-CoA over binding of pyruvate and acetyl-CoA. Interestingly, a close contact (2.4 Å) between the α-carbon of acetyl-CoA and the keto-carbon of pyruvate was reported for HaloMS [23]. Pyruvate and acetyl-CoA are actually substrates for the homologous malate synthase of *H. marismortui* (81% identity to the *H. volcanii* enzyme); the enzyme is able to catalyze the formation of (*S*)-citramalyl-CoA, but not the hydrolysis of its CoA thioester bond [15]. Although the *K*_m_-value for pyruvate was high (30 mM) for the *H. marismotui* malate synthase, the pyruvate concentration during soaking of the *H. volcanii* crystals was even higher, 70 mM [23]. We used the deposited structure factors for the HaloMS structure with bound pyruvate and acetyl-CoA (PDB 3OYZ) to re-examine the electron density. Interestingly, in the original maps there was still some additional positive density (*F*_O_-*F*_C_) between pyruvate and acetyl-CoA [23], indicating that there may be a connection of the carbon backbones of both substrates. Hence, we tried modeling in (*S*)-citramalyl-CoA. Superposition of the refined (*S*)-citramalyl-CoA (Additional file 3: Figure S3) shows that it fits slightly better into a simulated annealing omit map. This suggests that the carbon-carbon bond formation can still be catalyzed in these crystals and that there was probably a mixture of different reaction states present.

It should also be noted that both HaloMS as well as HaloMCL can act as malyl-CoA thioesterases, meaning that they are able to use malyl-CoA as substrate, in contrast to the canonical malate synthases [35,38]. Moreover, both haloarchaeal enzymes also release malyl-CoA as the product of acetyl-CoA and glyoxylate condensation during catalysis [15]. This is not the case with conventional malate synthases; they do not release malyl-CoA as an intermediate. Both the HaloMS and HaloMCL represent interesting chimeric enzymes combining MCL and malate synthase functions. Both are able to catalyze the same reactions as other MCLs, namely the formation and cleavage of malyl-CoA, β-methylmalyl-CoA, and (*S*)-citramalyl-CoA. However, they can also catalyze the hydrolysis of the malyl-CoA thioester bond like the other malate synthases, but they do not hydrolyze the CoA thioester bonds of β-methylmalyl-CoA or (*S*)-citramalyl-CoA. Although, HaloMS and HaloMCL have very similar substrate spectra and reaction specificities, the *K*_m_ values for the respective substrates differ significantly, defining their distinct functions in the methylaspartate cycle [15] as malate synthase or β-methylmalyl-CoA lyase, respectively.

Similarly, genome analysis revealed that there are two different types of MCLs present in *M. extorquens* that are regulated differentially, depending on the mode of growth [39]. One of the MCLs is phylogenetically related to the enzyme of *R. sphaeroides*, the other to the *C. auranticus* enzyme (Figure 2). It was found that *M. extorquens* also uses the ethylmalonly-CoA pathway to assimilate the acetyl-CoA that derives from its serine cycle [40,41] and it is also able to grow on acetate as the sole carbon source instead of C_1_ substrates by using the ethylmalonyl-CoA pathway. It is tempting to speculate that one of the MCLs in *M. extorquens* is specifically used for the cleavage of malyl-CoA in the serine cycle, whereas the primary functions of the second MCL are the cleavage of β-methylmalyl-CoA and the synthesis of malyl-CoA in the ethylmalonyl-CoA pathway. Both these enzymes may be optimized to work in one or the other direction under physiological substrate concentrations.

Two other distinct groups of the CitE-like superfamily of enzymes (type I and type II in Figure 2) may be carbon-carbon bond lyases as well. It was suggested that the CitE-like enzymes of *Mycobacterium tuberculosis* and *Yersinia pestis*, which belongs to the type I enzymes in Figure 2, also use free CoA thioesters as substrates [7,24]. Several more crystal structures of CitE-like superfamily enzymes are available although their physiological functions are unknown. Some of these structures were claimed to be the CitE subunit of citrate lyase, but that may not be correct, judging by amino acid sequence and phylogenetic analysis.

## Conclusions

Despite the relatively low amino acid sequence identity between MCLC and MCLR, their tertiary and quaternary structures are almost identical. The only prominent differences were found in the N-termini, which differ in size and orientation. These N-termini influence the strength of the interaction at the dimer interface between trimers in the hexameric assemblies. Furthermore, the comparison of the two MCL structures to the structures of HaloMS, MSG, and MSA does not explain why the MCLs are not able to hydrolyze CoA thioester bonds. Very few differences were observed between the active sites. Nevertheless, some hints emerge to explain the different substrate specificities of the MCLs compared to the different kinds of malate synthases. The structures and the previously proposed reaction mechanism for malate synthases suggest that malyl-CoA lyases should be able to hydrolyze CoA thioester bonds too. This however, has not been observed.

Collectively, these structures and the comparative analyses of the catalytic mechanism proposed for malate synthases lay the foundation for further studies including site directed mutagenesis to gain insights into the specific determinants of the different reaction specificities. Expanding the biochemical and structural knowledge about other CitE-like enzymes may also help to understand why MCLs are not hydrolyzing CoA-thioester bonds, especially since the structures of several CitE-like enzymes have been solved already but without knowledge of their respective functions, substrate spectra, and catalyzed reactions.

## Methods

### Cloning and protein expression

The gene coding for MCLC from *C. aurantiacus* OK-70-fl was amplified using chromosomal DNA as template. Two oligonucleotides (introduced restriction sites are italic) were designed upstream (5′- gggagaag*ca tatg*cgcaag ctagctc -3′; *Nde*I) and downstream (5′- gcgctcatcc ctct*aagctt* gctgcac -3′; *Hind*III) of the gene coding for MCLC. PCR was performed with *Pfu* polymerase for 32 cycles, including denaturation for 60 s at 94°C, annealing for 60 s at 58°C, and polymerization for 140 s at 72°C. The PCR product was cloned into the pT7-7 vector [42] for expression in *Escherichia coli* resulting in plasmid pT7-MCL_Ca.

The cloning of the MCLR from *R. sphaeroides* 2.4.1 was described by Erb *et al*. [11]. The plasmid pMCL1_RS_JZ_03 for overexpression in *E. coli* was kindly provided by Prof. Birgit Alber.

Competent *E. coli* BL21(DE3) cells were transformed with the respective plasmids and 1 liter cultures were grown at 27°C in of LB medium with 100 μg ampicillin ml^-1^. Due to the leaky expression of the plasmids, the cultures were not induced with IPTG. The cells were harvested after 24 h of growth and stored at -80°C until use.

The cloning and expression of the helper enzyme mesaconyl-C1-CoA hydratase was described previously [43].

### Purification of recombinant enzymes

All purification steps were performed at 4°C. Protein concentrations were determined using the Bradford method [44]. *E. coli* cells containing the recombinant MCLC were resuspended in a two-fold volume of 50 mM Tris(hydroxymethyl)aminomethane (Tris)/HCl buffer (pH 7.5) containing 2 mM of MgCl_2_. Cells were lysed by sonication (model W-220 F, Branson) and the lysate was heat precipitated for 15 min at 65°C followed by 40 min centrifugation (40,000 × *g*) at 4°C. A 7.5 ml DEAE fast flow Sepharose (Sigma-Aldrich, St. Louis, MO, USA) column was equilibrated with 20 mM Tris/HCl pH 7.5 containing 2 mM MgCl_2_ (buffer A). The supernatant of the heat precipitation was applied to the column at a flow rate of 1 ml min^-1^. The column was extensively washed with buffer A. The concentration of NaCl in buffer A was increased in 50 mM steps and MCLC eluted at 100 mM NaCl. The eluate was concentrated using centrifugal ultra-filtration devices with a molecular weight cut-off of 30 kDa (Amicon Ultra-15, Millipore, Billerica, MA, USA). A 24 ml gel filtration column (Superdex 200 10/30 GL, GE Healthcare, Waukesha, WI, USA) was equilibrated with 20 mM Tris/HCl pH 7.5 buffer containing 2 mM MgCl_2_ and 100 mM NaCl (buffer B). Concentrated MCLC was then applied to the column at a flow rate of 0.4 ml min^-1^.

*E. coli* cells containing N-terminal His_10_-tagged MCLR were suspended in a two-fold volume of 50 mM Tris/HCl pH 7.5, 250 mM NaCl and 5 mM MgCl_2_ (buffer C). Cells were lysed by sonication and the lysate was centrifuged for 40 min (40,000 × *g*) at 4°C. A 1 ml Ni-Sepharose column (HisTrap HP; GE Healthcare, Waukesha, WI, USA) was equilibrated with buffer C. The cell extracts (40,000 × *g* supernatants) were applied to the column at a flow rate of 1 ml min^-1^. The column was washed with buffer C containing 100 mM imidazole to remove unspecifically bound proteins. Recombinant His-tagged MCLR was eluted at 500 mM imidazole in buffer C. The enzyme was concentrated as described above and then applied to a 24 ml gel filtration column equilibrated with buffer B at a flow rate of 0.4 ml min^-1^.

The purification of the helper enzyme mesaconyl-C1-CoA hydratase was described previously [43].

Purified enzymes were concentrated and stored at 4°C for 2 weeks at most or at -80°C for several months before use. Protein standards used during gel filtration were thyroglobulin (670 kDa), γ-globulin (158 kDa), ovalbumin (44 kDa), myoglobin (17 kDa), and vitamin B12 (1.35 kDa).

### Enzyme activity assays

One unit (U) corresponds to an enzyme activity of 1 μmol min^-1^ mg_(protein)_^-1^. The activity of purified MCLC and MCLR was routinely confirmed in a previously described [10] coupled spectrophotometric assay that was slightly modified. In this assay the formation of β-methylmalyl-CoA from propionyl-CoA and glyoxylate is monitored. β-Methylmalyl-CoA is dehydrated by the coupling enzyme mesaconyl-C1-CoA hydratase, which can be followed at 290 nm. An estimated absorption coefficient of 3,400 M^-1^ cm^-1^ at 290 nm for the product mesaconyl-C1-CoA was used. This estimation is based on the assumption that the molar absorption coefficients at 260 nm (ϵ_260 nm_) of α,β-unsaturated CoA esters is 22,600 M^-1^ cm^-1^[45]. The assay mixture (0.4 ml) contained 200 mM MOPS/KOH buffer (pH 7.5), 5 mM MgCl_2_, 0.3 mM propionyl-CoA, 3 mM glyoxylate, 10 U of mesaconyl-C1-CoA hydratase, and recombinant MCLC or MCLR. The reaction was carried out at 30°C and was started by addition of either glyoxylate or MCL.

The condensation of pyruvate and acetyl-CoA to (*S*)-citramalyl-CoA by MCLR was observed in an HPLC based assay. The reaction mixture (0.5 ml) contained 200 mM MOPS/KOH (pH 7.5), 5 mM MgCl_2_, 0.5 mM acetyl-CoA, excess (100 mM) of pyruvate, and 0.25 U (referring to the formation of β-methylmalyl-CoA) of recombinant MCLR. After 0, 5 and 10 min of incubation a sample of 100 μl was withdrawn and the reaction was stopped on ice by addition of 10 μl of 90% formic acid. Precipitated protein was removed by centrifugation, and the supernatants analyzed for CoA thioesters by reversed phase HPLC.

### Analytical high-performance liquid chromatography (HPLC)

HPLC was performed using a Waters Alliance e2695 system (Waters, Milford, MA). Reaction products and standard compounds were detected by UV absorbance with a Waters 998 photodiode array detector at 260 nm. CoA thioesters were identified by retention times and their respective UV spectra (220 – 340 nm) as described elsewhere [10]. A reversed phase C_18_ column (Waters SymmetryShield, 4 μm, 250 × 4 mm) was used. A flow rate of 0.6 ml min^-1^ and a gradient of 28 min from 4 to 28% acetonitrile in 40 mM K_2_HPO_4_/HCOOH buffer (pH 4.2) were applied.

### Crystallization and structure determination

Crystals were grown at 22°C using either the sitting- or hanging-drop vapor diffusion methods. Two different approaches were used to grow crystals of MCLC: **(i)** Purified enzyme (3.5 mg ml^-1^) was mixed with 60 mM bis-Tris propane/citric acid pH 6.6, 20% (w/v) polyethyleneglycol (PEG) 3350, 20 mM MgCl_2_ in a ratio of 1:1 (enzyme:crystallization buffer). Crystals were briefly soaked with crystallization buffer supplemented with 25% (v/v) glycerol before the crystals were plunged into liquid nitrogen for freezing. **(ii)** Purified enzyme (7.5 mg ml^-1^) was mixed with 0.1 M Na-cacodylate pH 5.5, 20% (w/v) PEG 4 K and with buffer B containing 20 mM propionyl-CoA and 25 mM sodium-oxalate (buffer B-PO) in a ratio of 1:2:1 (enzyme:crystallization buffer:buffer B-PO). Crystal drops were supplemented with 20% (v/v) PEG 400 shortly before the crystals were cryo-cooled in liquid nitrogen.

Likewise, two different conditions were used to grow crystals of MCLR: **(i)** Purified enzyme (3 mg ml^-1^) was mixed with 0.1 M Tris/HCl pH 8.5, 20% (w/v) PEG 3350, 20 mM MgCl_2_ in a ratio of 2:3 (enzyme:crystallization buffer). Crystals were soaked for varying time periods in crystallization drop mixture supplemented with 8 mM propionyl-CoA and 25% (v/v) glycerol before the crystals were plunged into liquid nitrogen for freezing. **(ii)** Purified enzyme (2.5 mg ml^-1^) was mixed with 4-(2-hydroxyethyl)-1-piperazineethanesulfonic acid (HEPES)/NaOH pH 7.5, 0.1 M MgCl_2,_ 10% (w/v) PEG 4000 and with buffer B-PO in a ratio of 1:2:1 (enzyme:crystallization buffer:buffer B-PO). Crystal drops were supplemented with 20% (v/v) PEG 400 shortly before the crystals were cryo-cooled in liquid nitrogen.

X-ray diffraction data were collected at the Lawrence Berkeley Laboratory Advanced Light Source (beamlines 5.0.1, 5.0.2, 5.0.3). The data was processed with XDS [46] and the CCP4 software package [47]. All structures were solved by molecular replacement using AutoMR-, Phaser-MR-, and AutoBuild-programs of the Phenix software package [48]. The MCLR structure was solved first using the structure of a CitE-like enzyme from *Mycobacterium tuberculosis* (PDB 1U5H) [7] as the search model. The structure of MCLR was refined with Phenix.refine and subsequently used as a search model to solve the structure of MCLC. Additional manual modeling and ligand fitting was done with COOT [49]. Further refinements, as well as water-picking for all structures were performed by Phenix.refine. The atomic coordinates and structure factors (PDB IDs: 4L7Z, 4L80, 4L9Y, 4L9Z) have been deposited in the Protein Data Bank [http://wwpdb.org/].

### Phylogenetic tree construction

Amino acid sequence searches within the domains of Bacteria and Archaea were performed using BLAST [http://blast.ncbi.nlm.nih.gov/]. Accession numbers for reference sequences are provided in Table 1. A cut-off E value of 1e^-60^ was applied for all BLAST searches except for those using the β-subunit of citrate lyase of *Klebsiella pneumonia* as query in which the cut-off was 1e^-80^. All sequences that appeared to be truncated were removed from the data set. All sequences that were more than 90% identical to another one were removed with CD-HIT [50] to further reduce the amount of data. The sequences of the *E. coli* malate synthases A and G (Accession AAC76984 and AAC76012, respectively) were added and alignment was performed with the BioEdit program [http://www.mbio.ncsu.edu/bioedit/bioedit.html] using the similarity matrix PAM250. The phylogenetic tree was constructed from a total of 675 amino acid sequences using MEGA5 [51] and the Neighbor-Joining method [52]. A bootstrap consensus tree [53] was inferred from 1000 replicates. The distances were calculated using the Poisson method. Positions in the alignment with less than 95% coverage were eliminated.

### Other methods

Chimera [54] was used to analyze and align crystal structures and to make figures. Protein interfaces were analyzed using the PISA webserver [55] [http://www.ebi.ac.uk/msd-srv/prot_int/pistart.html]. Protein-protein interactions were also analyzed by use the PIC-webserver tool [56] [http://pic.mbu.iisc.ernet.in]. Hidden Markov models (HMM) and HMM logos were created using HMMER Version 3.0 [57] and LogoMat-M [58], respectively. The structures were also analyzed by PDBsum [59].

## Competing interest

The authors declare that they have no competing interest.

## Authors’ contributions

JZ and CAK designed the research. JZ produced and crystallized the enzymes, collected and processed the diffraction data, and solved, modeled and refined the structures. JZ and CAK analyzed the structures. JZ and CAK wrote the paper. Both authors read and approved the final manuscript.

## Supplementary Material

Additional file 1: Figure S1 HMM–Logo of the amino acid sequences of enzymes that cluster together with MCLC in the phylogenetic tree. Numbering of residues corresponds to MCLC. The 48 sequences comprised in this figure share at least 57% sequence identity. Catalytic residues are marked with asterisks. Residues that coordinate the Mg^2+^ ion are labeled. The region responsible for the movement of the C-terminal lid domain is marked “bending”. Secondary structure elements derived from a PDBsum analysis of the MCLC structure (PDB 4L80) are aligned with the HMM-logo. Click here for file

Additional file 2: Figure S2 HMM-Logo of the amino acid sequences of enzymes that cluster together with MCLR in the phylogenetic tree. The numbering of residues corresponds to MCLR. Only sequences were used (93 sequences in total) that share at least 50% sequence identity to MCLR. Catalytic residues are marked with asterisks. Residues that coordinate the Mg^2+^ ion are labeled. The region responsible for the movement of the C-terminal lid domain is marked “bending”. Secondary structure elements derived from a PDBsum analysis of the MCLR structure (PDB 4L9Z) are aligned with the HMM-logo. Click here for file

Additional file 3: Figure S3 Superpositions of Fo-Fc electron density simulated annealing omit maps on refined ligands for the malate synthase of H. volcanii. A) Omit map at 2.5 σ for acetyl-CoA and pyruvate. The α-carbon of the acetyl moiety is in very close proximity to pyruvate (2.4 Å). B) Omit map at 2.5 σ for (*S*)-citramalyl-CoA. The position of the β-carbon of citramalyl-CoA (formerly keto-carbon of pyruvate) is slightly shifted and its bonds assume a tetrahedral geometry compared to the planar geometry of pyruvate in A. Click here for file
