# Supplementary figures and images for: The crystal structures of the tri-functional Chloroflexus aurantiacus and bi-functional Rhodobacter sphaeroides malyl-CoA lyases and comparison with CitE-like superfamily enzymes and malate synthases
Source: BMC Struct Biol. 2013 Nov 9;13:28. doi: 10.1186/1472-6807-13-28 (PMC3832036; doi:10.1186/1472-6807-13-28)

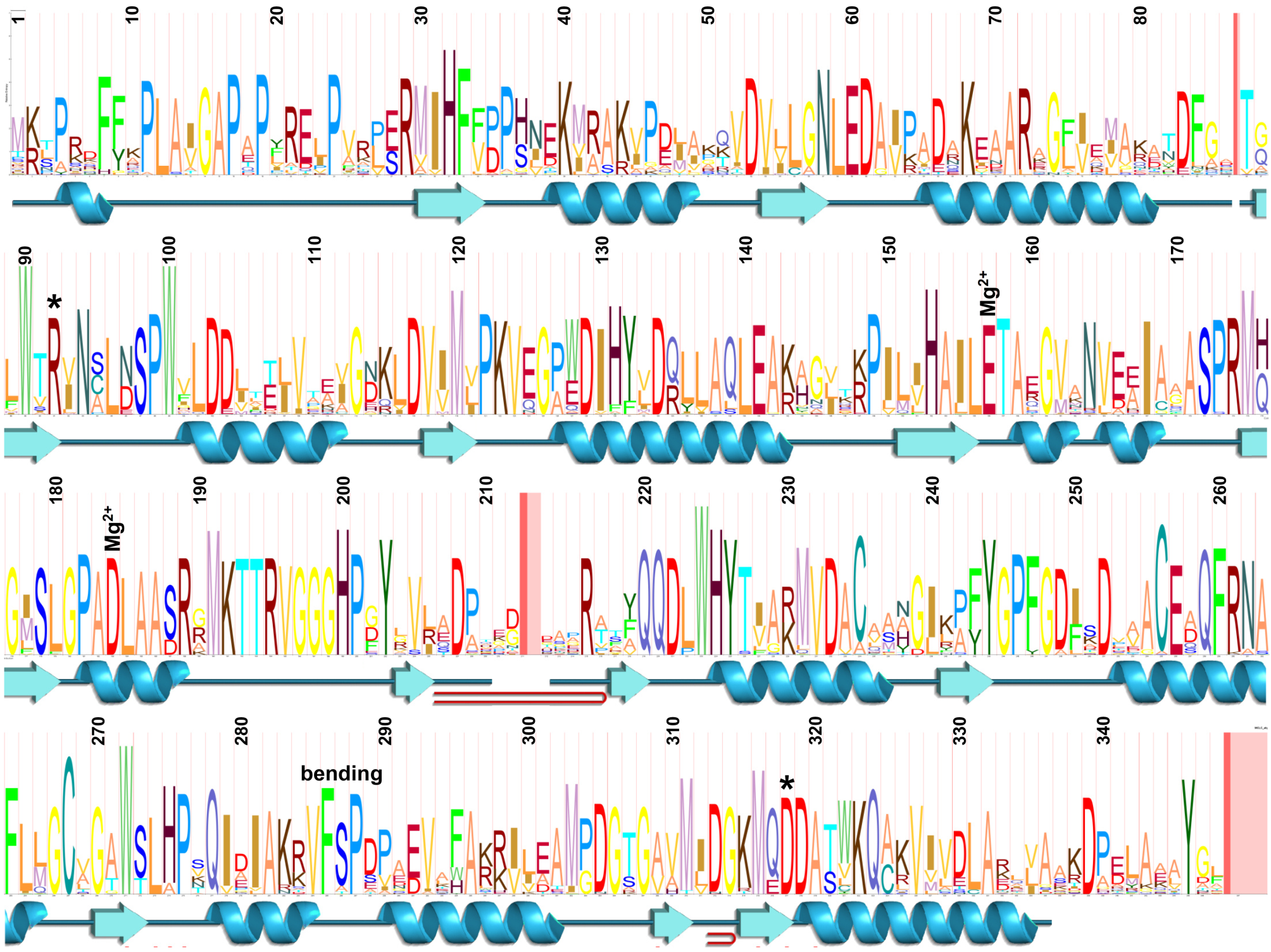

Supplement: Additional file 1: Figure S1 — HMM–Logo of the amino acid sequences of enzymes that cluster together with MCLC in the phylogenetic tree. Numbering of residues corresponds to MCLC. The 48 sequences comprised in this figure share at least 57% sequence identity. Catalytic residues are marked with asterisks. Residues that coordinate the Mg2+ ion are labeled. The region responsible for the movement of the C-terminal lid domain is marked “bending”. Secondary structure elements derived from a PDBsum analysis of the MCLC structure (PDB 4L80) are aligned with the HMM-logo. [file 1472-6807-13-28-S1.tiff]

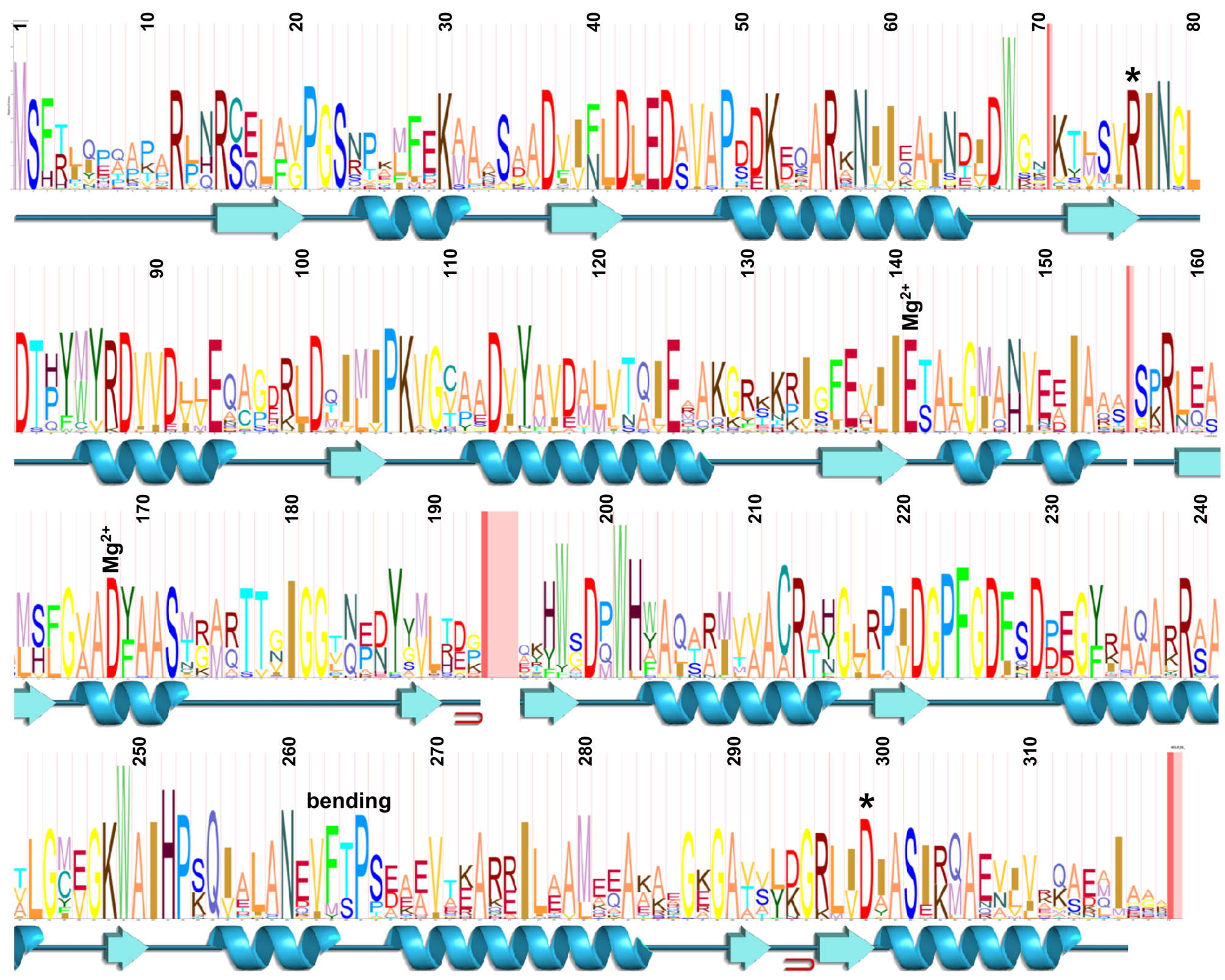

Supplement: Additional file 2: Figure S2 — HMM-Logo of the amino acid sequences of enzymes that cluster together with MCLR in the phylogenetic tree. The numbering of residues corresponds to MCLR. Only sequences were used (93 sequences in total) that share at least 50% sequence identity to MCLR. Catalytic residues are marked with asterisks. Residues that coordinate the Mg2+ ion are labeled. The region responsible for the movement of the C-terminal lid domain is marked “bending”. Secondary structure elements derived from a PDBsum analysis of the MCLR structure (PDB 4L9Z) are aligned with the HMM-logo. [file 1472-6807-13-28-S2.tiff]

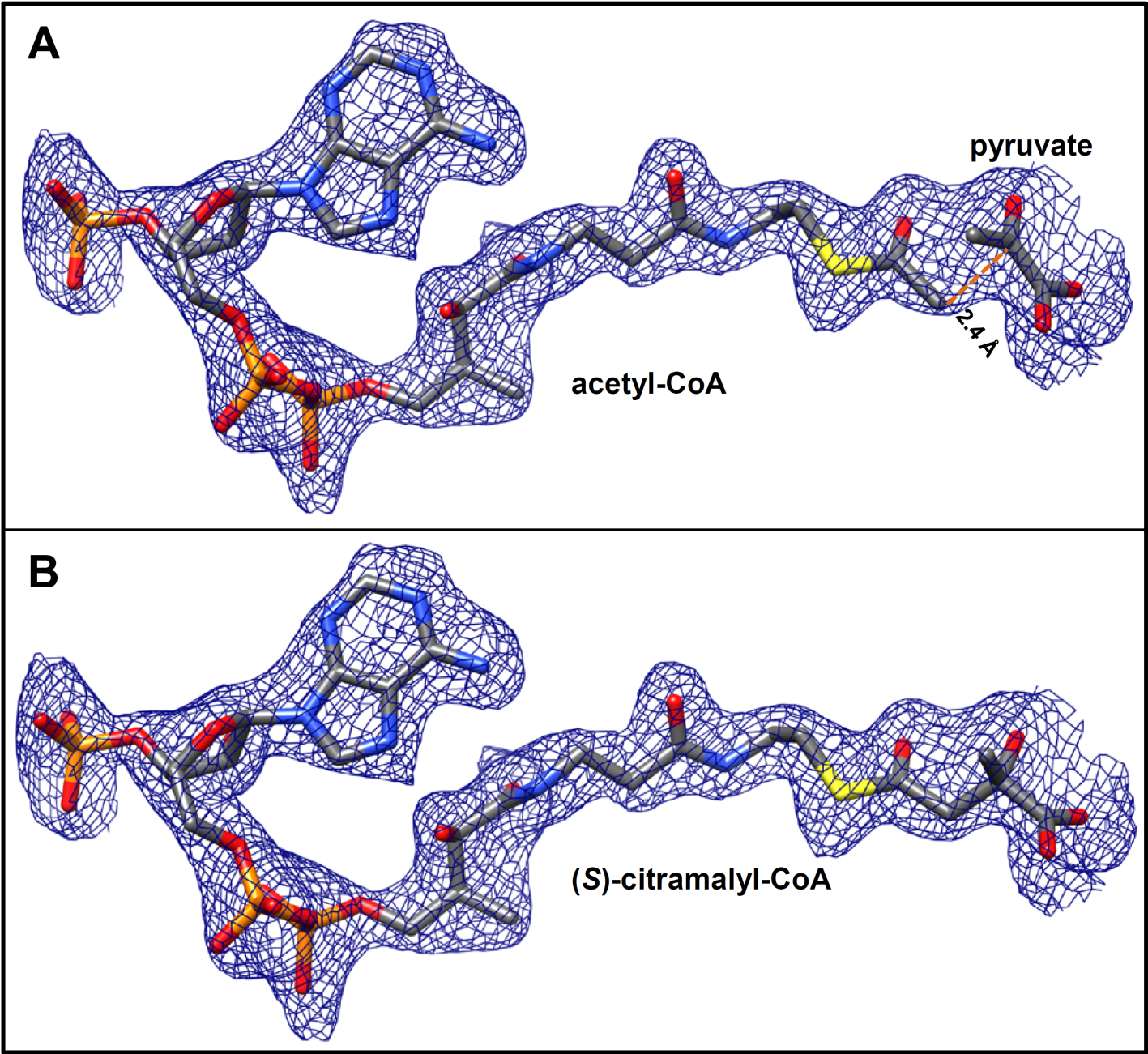

Supplement: Additional file 3: Figure S3 — Superpositions of Fo-Fc electron density simulated annealing omit maps on refined ligands for the malate synthase of H. volcanii. A) Omit map at 2.5 σ for acetyl-CoA and pyruvate. The α-carbon of the acetyl moiety is in very close proximity to pyruvate (2.4 Å). B) Omit map at 2.5 σ for (S)-citramalyl-CoA. The position of the β-carbon of citramalyl-CoA (formerly keto-carbon of pyruvate) is slightly shifted and its bonds assume a tetrahedral geometry compared to the planar geometry of pyruvate in A. [file 1472-6807-13-28-S3.tiff]
